# Case report: High-dose epoprostenol therapy in pediatric patients with pulmonary hypertension and developmental lung disease

**DOI:** 10.3389/fped.2023.1116434

**Published:** 2023-03-03

**Authors:** Yoshie Fukasawa, Hidenori Yamamoto, Miharu Ito, Akiko Saito, Kiyotaka Go, Yoshihito Morimoto, Kazushi Yasuda, Yoshiaki Sato, Masahiro Hayakawa, Taichi Kato

**Affiliations:** ^1^ Department of Pediatrics, Nagoya University Graduate School of Medicine, Nagoya, Japan; ^2^ Division of Neonatology, Center for Maternal-Neonatal Care, Nagoya University Hospital, Nagoya, Japan; ^3^ Second Department of Pediatrics, Ogaki Municipal Hospital, Ogaki, Japan; ^4^ Department of Pediatrics, Japanese Red Cross Aichi Medical Center Nagoya Daiichi Hospital, Nagoya, Japan; ^5^ Department of Pediatric Cardiology, Aichi Children's Health and Medical Center, Obu, Japan

**Keywords:** pulmonary hypertension, developmental lung disease, epoprostenol, congenital diaphragmatic hernia, alveolar capillary dysplasia with misalignment of pulmonary veins, bronchopulmonary dysplasia

## Abstract

Pulmonary hypertension (PH) with developmental lung disease is a life-threatening disease and accounts for 10%–12% of pediatric PH patients. Administration of specific pulmonary vasodilators to pediatric PH patients has brought about improvement of their long-term prognosis. Intravenous epoprostenol therapy is a gold standard therapy for severe idiopathic pulmonary arterial hypertension (IPAH), but there are few reports demonstrating the efficacy of epoprostenol for pediatric PH patients with developmental lung disease, especially when treating with high doses of epoprostenol. Two cases of pediatric PH patients with alveolar capillary dysplasia with misalignment of pulmonary veins (ACD/MPV) and congenital diaphragmatic hernia (CDH) with bronchopulmonary dysplasia (BPD), respectively, treated with epoprostenol above 100 ng/kg/min are presented. In these two cases, severe PH was improved significantly by an aggressive increase of the epoprostenol infusion rate with administration of oral pulmonary vasodilators and appropriate respiratory management, without any significant adverse effects. High-dose epoprostenol therapy may be one of the therapeutic options in pediatric PH patients with developmental lung disease.

## 1. Introduction

Pulmonary hypertension (PH) is a life-threatening disease characterized by sustained elevation of pulmonary artery pressure (PAP) resulting from progressive pulmonary vasoconstriction and vascular remodeling. PH categorized as group III, which is classified as “PH due to lung diseases and/or hypoxia”, accounted for 10%–12% for pediatric PH patients in a past registry (1), and the number of patients has been markedly increasing in recent years (2).

Prostaglandin analogs, phosphodiesterase type 5 (PDE5) inhibitors, and endothelin receptor antagonists (ERAs) are available for the treatment of PH and have been shown to contribute to improvement of its long-term prognosis. Of them, the effectiveness of intravenous epoprostenol therapy was demonstrated in past retrospective studies of pediatric PH patients (3, 4), and it is one of the gold standard therapies for severe PH (5). It has been demonstrated that high-dose epoprostenol at more than 100 ng/kg/min could be administered to adult patients with idiopathic pulmonary arterial hypertension (IPAH) without any severe adverse effects (6, 7), resulting in lower mean pulmonary artery pressure (mPAP) and pulmonary vascular resistance (PVR) compared with the previous recommended dose (8).

There have been few reports of high-dose epoprostenol administered to pediatric PH patients with developmental lung disease, especially early infants (9). In this report, two cases of early infants with group III PH caused by developmental lung disease who were administered high-dose epoprostenol at above 100 ng/kg/min without any significant adverse effects are reported.

## 2. Case description

### 2.1. Case 1

**Figure 1:**
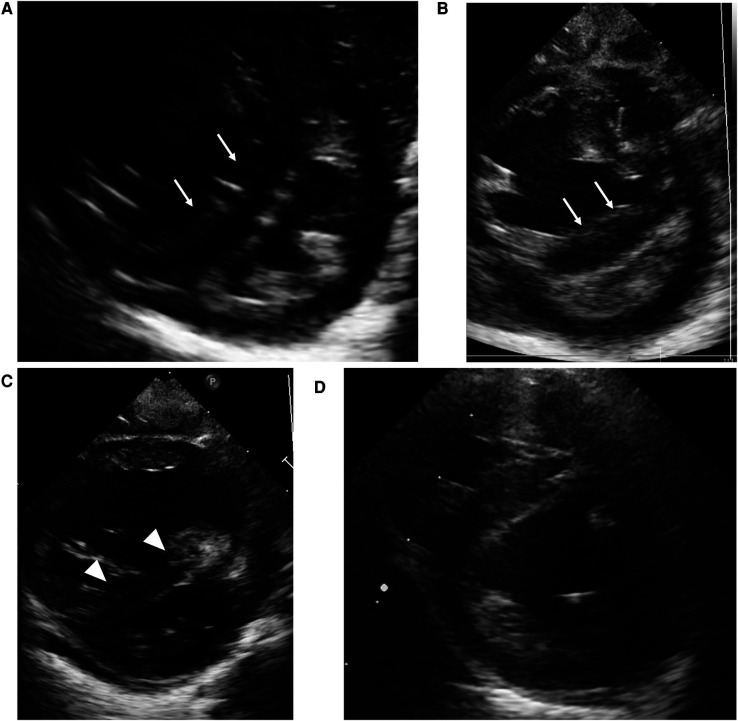
Case 1: echocardiography in the parasternal short axis view. Echocardiography at birth (**A**) and at the time of PH crisis (**B**) showing septal bowing to the left (white arrow) and a D-shaped left ventricle that indicates over-systemic pulmonary arterial pressure. On the day after the initiation of high-dose epoprostenol (**C**), left sided deviation of intraventricular septum (white arrowhead) ameliorated compared to at the time of PH crisis. At one year of age (**D**), the interventricular septal shift and left ventricle compression have improved.

**Figure 2:**
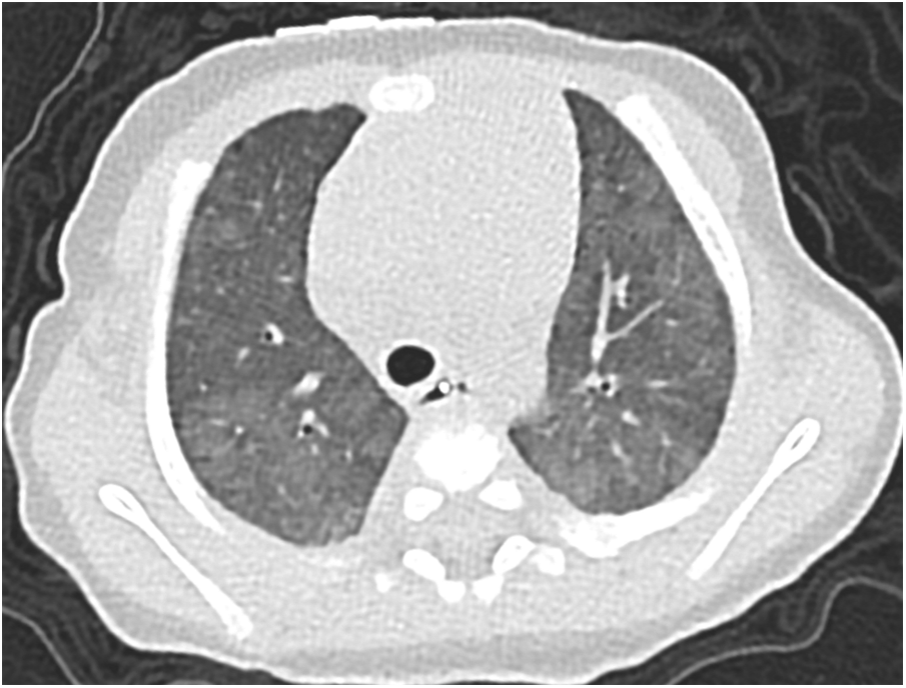
Case 1: axial CT images of lung window settings at 3 months. Axial CT images show heterogeneous ground-glass opacities in both lung fields.

A 37-week-gestational age male neonate with an omphalocele and duodenal stenosis, weighing 2,506 g, was delivered by elective cesarean section, with an Apgar score of 8 and 9 at 1 and 5 min, respectively. He showed retractive breathing and tachypnea at birth but did not require oxygen inhalation. The chest radiograph at birth showed bilateral fine granular opacification. Echocardiography at birth (Figure 1A) showed no structural anomaly, a flattened interventricular septum, and a bidirectional patent ductus arteriosus (PDA) shunt, which indicated severe PH. At one day of age, just before the scheduled operation for duodenal stenosis, hypoxemia caused by PH appeared, and he was then intubated. PH was apparently exacerbated after the operation because echocardiography suggested that pulmonary arterial pressure (PAP) was greater than systemic arterial pressure, so inhaled nitric oxide (NO) and intravenous epoprostenol up to 40 ng/kg/min were administered. Prostaglandin E1 was administered for run off between 2 and 4 days of age. Because severe PH associated with respiratory failure persisted, chest computed tomography (CT) was performed at 1 month of age, and it showed ground-glass opacities in both lung fields (Figure 2). Serum pulmonary surfactant protein-D was elevated (406.0 ng/ml). Genetic analysis for interstitial lung disease was submitted. Lung biopsy and bronchoalveolar lavage were not performed due to the high risk of PH crisis. The diagnostic workup was summarized in Supplementary Table S1. Considering interstitial lung disease based on the CT images, steroid pulse therapy was given. After this treatment, NO and epoprostenol were discontinued about 2 months after surgery, with the addition of oral tadalafil and macitentan. PAP almost equal to systemic arterial pressure persisted on echocardiography.

At three months of age, 2 days after administration of inactivated vaccines, severe desaturation of oxygen triggered by crying appeared. The echocardiogram (Figure 1B) demonstrated septal bowing to the left and a compressed left ventricle, which indicated PAP greater than systemic arterial pressure. Re-administration of epoprostenol with inhalation of high concentrations of oxygen and NO along with intravenous sedation against PH crisis were immediately started. Desaturation and hypotension due to PH persisted even with 100% oxygen but improved with rapid intravenous epoprostenol infusion followed by high-dose continuous infusion at 120 ng/kg/min. Echocardiography on the day after the initiation of high-dose epoprostenol (Figure 1C) showed an improvement in the deviation of intraventricular septum compared to at the time of PH crisis. He presented with flushed skin, irritability, and sleep disturbance, but he did not show any severe adverse effects, such as hypotension, bradycardia, or pulmonary edema. After the infusion rate was reduced to 85 ng/kg/min, the adverse effects, except for skin redness, were ameliorated. At 6 months of age, heterozygous deletion in *FOXF1* enhancer region was detected by multiplex ligation-dependent probe amplification (MLPA), and he was diagnosed with alveolar capillary dysplasia with misalignment of pulmonary veins (ACD/MPV). PH crises triggered by infection or vaccination occurred several times, and thus the epoprostenol infusion rate was maintained in the range of 70–110 ng/kg/min for about 8 months without any other significant adverse effects. At 10 months of age, he was converted from intravenous epoprostenol to intravenous treprostinil in a stepwise manner. Echocardiography (Figure 1D) showed that the interventricular septal shift and LV compression improved, and right heart catheterization showed moderate pulmonary arterial hypertension (PAH) with a PAP of 52/26 mmHg (mean, 39 mmHg), a systolic pulmonary systemic arterial pressure ratio (Pp/Ps) of 0.71, and a PVR index of 4.4 Wood units-m^2^ on the intravenous treprostinil infusion (125 ng/kg/min), oral tadalafil, and macitentan at one year of age. After switching from an intravenous infusion of treprostinil to subcutaneous infusion, he was discharged from the hospital at the age of one year and 6 months. It was necessary to continue PH treatment with 3 pulmonary vasodilators until lung transplantation. He underwent a lung transplant from a brain-dead donor at 3 years of age and is alive around a year after transplant.

### 2.2. Case 2

A full-term male neonate with a right congenital diaphragmatic hernia (CDH) was born by elective cesarean section, weighing 3144 g, with an Apgar score of 4 and 4 at 1 and 5 min, respectively. Immediately after birth, he underwent intubation for mechanical ventilation, but hypoxemia and ventilatory failure with severe PH were uncontrolled, which required venovenous extracorporeal membrane oxygenation support for 5 days combined with inhaled NO and infusion of intravenous epoprostenol. Following patch closure of the CDH at the age of five days, the PH gradually ameliorated, and it was confirmed that his PAP was almost normal without any selective pulmonary vasodilator on echocardiography at 3 months of age. Nevertheless, the attempts to wean him from mechanical ventilation were unsuccessful because of right lung hypoplasia, bronchomalacia, and bronchopulmonary dysplasia (BPD), which were found on bronchoscopy and chest CT. Accordingly, he underwent tracheostomy to allow long-term respiratory support at 6 months of age.

**Figure 3:**
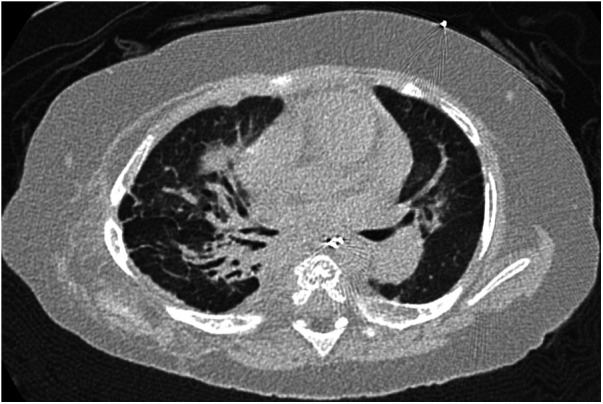
Case 2: axial CT images of lung window settings at 1 year of age. Axial CT images show parahilar consolidation and subpleural linear opacities of atelectasis or fibrosis with bronchodilation, predominantly in the right lung field.

**Figure 4:**
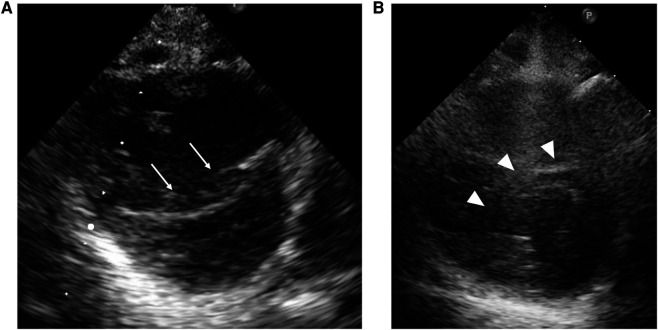
Case 2: echocardiography in the parasternal short-axis view. Echocardiography at the beginning of epoprostenol infusion (**A**) shows that the inverted interventricular septum (white arrow) compresses the left ventricle. At the time of epoprostenol infusion above 100 ng/kg/min (**B**), the interventricular septal shift (white arrowhead) and LV compression ameliorated.

At one year of age, he presented with low oxygen saturation, and it was necessary to gradually increase the oxygen concentration supplied to him. Chest CT images (Figure 3) showed para-hilar consolidation and subpleural linear opacities predominantly in the right lung field, indicating atelectasis or fibrosis. The diagnostic workup was summarized in Supplementary Table S1. Aspiration pneumonia and atelectasis due to gastroesophageal reflux (GER) were thought to contribute to the exacerbation of BPD, and Nissen fundoplication was performed. Preoperative echocardiography showed a flattened intraventricular septum, which suggested severe PH caused by BPD. Since it was predicted that operative stress would result in deterioration of the PH, epoprostenol was restarted at an initial rate of 4 ng/kg/min with the patient under continuous deep sedation with a muscle relaxant after the operation. Echocardiography after operation (Figure 4A) showed that the inverted intraventricular septum compressed the left ventricle, and the estimated systolic PAP (sPAP) based on Doppler assessment of the tricuspid regurgitant pressure gradient was around 95 mmHg. When he was awakened one week after surgery, it was necessary to increase the epoprostenol to 60 ng/kg/min, and addition of inhaled NO (up to 20 ppm) resulted in an estimated sPAP of around 65 mmHg. After 17 days of NO therapy, the estimated sPAP was temporarily stabilized in the range of 40–50 mmHg, with the addition of oral tadalafil and macitentan. However, labored breathing resulting from exacerbation of BPD appeared 3 weeks following discontinuation of NO. As fractional inspired oxygen gradually increased over 2 months due to hypoxemia, estimated sPAP also increased to above 85 mmHg on echocardiography again. It was necessary to increase the epoprostenol from 36 ng/kg/min to 106 ng/kg/min over two months and add steroid for BPD, resulting in estimated sPAP of 50 mmHg and improvement of IVS shift towards LV on echocardiography (Figure 4B). Epoprostenol was maintained at 74 ng/kg/min for the next 4 months due to the chronic nature of PH with BPD, resulting in an estimated sPAP between 50 and 70 mmHg. He showed redness of the skin, predominantly of his face, and itching, but no severe adverse effects such as hypotension or bradycardia, when epoprostenol was given at above 70 ng/kg/min. He could tolerate the symptoms of both adverse effects with antihistamines.

## 3. Discussion

Two cases of the use of high-dose epoprostenol infusions at above 100 ng/kg/min in infants with group III PH classified as developmental lung disease were presented. Although the use of pulmonary vasodilators is controversial in adults with group III PH, the present cases suggested that epoprostenol therapy, even if at a high dose, may be useful in ameliorating PH classified as group III in children. As far as we know, this is the second case report of high-dose epoprostenol therapy at above 100 ng/kg/min in infants with PH associated with developmental lung disease (9). In the present two cases, their severe PH was significantly ameliorated following repeated exacerbations and remissions of PH by aggressive increases of the epoprostenol infusion rate with the administration of oral pulmonary vasodilators and appropriate respiratory management.

In adult PAH patients, multicenter, prospective, randomized trials showed that continuous intravenous epoprostenol administration decreased PVR and improved exercise tolerance and the survival rate (10). Therefore, recent guidelines recommended the administration of epoprostenol to patients with PAH (group I) classified as WHO functional classes III and IV (8). In pediatric PAH patients, no large-scale, randomized, controlled study of selective pulmonary dilators containing epoprostenol has been conducted, and treatment is generally based on expert opinion and small-scale observational studies (11). In pediatric patients, epoprostenol is mainly administered for severe PAH, as for adults, and some observational studies showed improvement of the survival rate and exercise tolerance (3, 4, 12, 13).

On the other hand, randomized, controlled trials in adult patients with group III PH due to BPD did not show the effect of specific pulmonary vasodilators (14–16), and there is no established medical therapy. Developmental lung disease in group III PH is mainly found in the neonatal period and infancy, which includes various diseases such as BPD, pulmonary hypoplasia associated with CDH, ACD/MPV, and surfactant dysfunction (17), and published reports on the effects of pulmonary vasodilators for each disease do not provide sufficient evidence (18). The present two cases suggest that some patients may show the effectiveness of high-dose epoprostenol for PH accompanied with ACD/MPV or CDH with BPD.

ACD/MPV is a rare and fatal developmental lung disease that typically presents with severe respiratory failure and PH within hours to days after birth (19). Past case reports of PH with ACD/MPV showed that specific pulmonary vasodilators such as inhaled NO, intravenous epoprostenol, and sildenafil are administered to reduce PVR. However, the effects of these drugs are typically temporary (9, 19–22).

In atypical ACD/MPV, which is mild clinical presentation or late onset, two case series showed all patients had partial or complete response to specific pulmonary vasodilators administered prior to lung transplantation (21, 22). In these reports, the pathological abnormalities of this disease such as thickening of interalveolar septa, reduction of pulmonary alveolar capillaries, and misaligned pulmonary veins were focal or patchy and a heterogeneous distribution. In these cases, the response to specific pulmonary vasodilator and the adverse effect of pulmonary edema did not correspond to the severity of the pathological findings (21, 22). Despite the effectiveness of specific pulmonary vasodilators to the lesions of medial hypertrophy of small pulmonary arteries and arterioles, comorbid capillary and post-capillary lesions would cause pulmonary edema and make treatment for PH with atypical ACD/MPV difficult. Case 1 had an atypical clinical course in ACD/MPV. We speculate that the lack of pulmonary edema during administration of specific pulmonary vasodilators may be due to mild capillary and post-capillary lesions.

PH associated with BPD (BPD-PH), especially in the setting of BPD and CDH, leads to a complicated course in infancy and increased late mortality, which is strongly associated with the severity of PH (23, 24). Regarding the effect of specific pulmonary vasodilators, several small-scale studies indicated that sildenafil, a PDE 5 inhibitor, was effective for infants with BPD-PH. Kadmon et al. reported that administration of sildenafil and/or bosentan improved PH associated with BPD in 78% of 20 infants, with a 2-year survival rate of 95%, compared to 52%–62% in previous reports (25). As for intravenous epoprostenol therapy for infants with BPD-PH, only a few reports demonstrated that intravenous epoprostenol was largely tolerable and ameliorated PH (26, 27). Although the present cases responded to high-dose epoprostenol without any significant adverse effects, further cases need to be studied to select patients who will benefit from epoprostenol, to properly monitor adverse effects, and to set treatment goals for both diseases.

According to the ESC/ERS guidelines for PH, epoprostenol is usually started at 2–4 ng/kg/min and then gradually increased to 20–40 ng/kg/min in the absence of adverse effects (8). For pediatric patients with PH, epoprostenol is often administered intravenously at a higher dose than for adult patients, and the appropriate dosage of epoprostenol ranges from 50 to 80 ng/kg/min (5, 11). Concerning the adverse effects of epoprostenol at the usual dose, flushing, cyanosis, and pain were observed less frequently in infants with PH under 1 year of age than in adults, and 2 of 21 infants exhibited bleeding at initiation and when epoprostenol was increased (28).

A high dose of epoprostenol exceeding 100 ng/kg/min improved mean PAP, PVR, and WHO functional class in adult patients with IPAH and hereditary pulmonary arterial hypertension (6, 29), and it improved the survival rate more in the rapid increase group than in the slow increase group (7). In infants with group III PH, there are only two case reports, and they showed that the epoprostenol dose was as high as 120 ng/kg/min and 96 ng/kg/min, respectively, without serious adverse effects (9, 30). In the present two cases, the dose of epoprostenol was rapidly increased and maintained at a high dose above 100 ng/kg/min under careful monitoring in the NICU, and the patients showed only tolerable adverse effects such as skin flushing skins, itching, irritability, and sleep disturbance. Although the present cases showed no serious adverse effects, close monitoring is required at the start of epoprostenol therapy and during the rapid increase of its dose due to concerns about hypotension and exacerbation of cyanosis due to V/Q mismatch and an increased right-to-left shunt in the lung when epoprostenol is administered intravenously for PH patients, especially with lung disease (18).

In pediatric BPD-PH patients, exposure of the pulmonary vessels to various injurious stimuli, which include oxygen free radicals, ventilator-induced lung injury, or inflammation, leads to the development and exacerbation of PH (31). Optimization of respiratory management and intervention for factors exacerbating lung disease are recommended for PH patients with developmental lung disease (11, 32). The present two cases required supplemental oxygen and artificial ventilation for a long period of time, and they had repeated respiratory infections, resulting in exacerbation of PH. They underwent some lung treatments such as steroid therapy, careful management of mechanical ventilation, and surgery for GER to prevent recurrent aspiration, in addition to pulmonary vasodilator therapy. Treatments to improve the lung condition in addition to administration of epoprostenol may also bring gradual improvement in PH.

In conclusion, high-dose epoprostenol therapy at above 100 ng/kg/min for two cases with PH associated with developmental lung disease was effective without any severe adverse effects. High-dose epoprostenol therapy may be one of the therapeutic options in pediatric patients with group III PH.

## Data Availability

The raw data supporting the conclusions of this article will be made available by the authors, without undue reservation.
