# Supplementary material for: Case report: High-dose epoprostenol therapy in pediatric patients with pulmonary hypertension and developmental lung disease
Source: Front Pediatr. 2023 Mar 3;11:1116434. doi: 10.3389/fped.2023.1116434 (PMC10020523; doi:10.3389/fped.2023.1116434)
Supplement: Supplementary file 1 [file Table1.docx]

|  | Case1 | Case2 |
| --- | --- | --- |
| Chest CT | Heterogeneous ground-glass opacity | Parahilar consolidation and subpleural linear opacities of atelectasis or fibrosis with bronchodilation |
| Lung biopsy | None | None |
| Bronchoalveolar lavage | None | None |
| Bronchoscopy | Tracheomalacia | Bilateral bronchomalacia  Tracheomalacia |
| Serum test  　KL-6  　SP-D | 155 U/ml  406.0 ng/ml | None  None |
| Chromosome test | 46, XY | 46, XY |
| Gene analysis | Heterozygous deletion in *FOXF1* enhancer region | None |

Supplementary table 1: Diagnostic workup for case 1 and 2

Bronchoalveolar lavage (BAL), Sialyrated glycoprotein Krebs von den Lungen 6 (KL-6), Serum pulmonary surfactant protein-D(SP-D)
